# Mass Spectrometry-Based Proteomics of Minor Species in the Bulk: Questions to Raise with Respect to the Untargeted Analysis of Viral Proteins in Human Tissue

**DOI:** 10.3390/life13020544

**Published:** 2023-02-15

**Authors:** Shahid Aziz, Faisal Rasheed, Rabaab Zahra, Simone König

**Affiliations:** 1 Patients Diagnostic Lab, Pakistan Institute of Nuclear Science and Technology (PINSTEC), Islamabad 44000, Pakistan; 2 Department of Microbiology, Faculty of Biological Sciences, Quaid-i-Azam University, Islamabad 45320, Pakistan; 3 IZKF Core Unit Proteomics, University of Münster, 48149 Münster, Germany

**Keywords:** viromics, mass spectrometry, gastric cancer, biopsy

## Abstract

**Simple Summary:**

Proteomics is a booming field in life sciences and, increasingly, is not only based on mass spectrometry (MS), but also on multiplex bead or aptamer assays and proximity extension assays. The use of untargeted MS generates big data sets in little time, but it also has limitations. Here, we discuss the plausibility of screening proteomic shotgun MS raw data for viral proteins in human gastric biopsies. Though this is technically possible and, thus, appealing to researchers, low-abundant proteins of guest species are barely present at suitable concentrations for measurement. Still, the processing algorithms will return hits due to chance assignments of low-quality spectra, but these should be red-flagged. A sanity check needs to establish whether or not certain proteins can be available at a sufficient concentration for measurement in the sample at all. Not every possible analysis is, thus, sensible. Even though both instrumentation and bioinformatic processing are continuously improving, a quality control of the data output will always be advisable. This paper uses practical examples to explain difficulties in spectral assignment leading to false-positive protein matches and is, thus, a tutorial for laymen and novices in MS-based proteomics.

**Abstract:**

(1) Background: Untargeted mass spectrometry (MS)-based proteomic analysis is highly amenable to automation. Software algorithms translate raw spectral data into protein information obtained by a comparison to sequence databases. However, the technology has limitations, especially for analytes measured at the limit of detection. In a protein expression study of human gastric biopsies, the question arose whether or not it is possible, as well as sensible, to search for viral proteins in addition to those from the human host. (2) Methods: Experimental data-independent MS data were analyzed using protein sequences for oncoviruses, and BLAST analyses were performed to elucidate the level of sequence homology to host proteins. (3) Results: About one hundred viral proteins were assigned, but there was also up to 43% sequence homology to human proteins. (4) Conclusions: There are at least two reasons why the matches to viral proteins should be used with care. First, it is not plausible that large amounts of viral proteins should be present in human gastric biopsies, so the spectral quality of the peptides derived from viral proteins is likely low. As a consequence, the number of false assignments is high. Second, homologous peptides found both in human and virus proteomes contribute to matching errors. Thus, though shotgun proteomics raw data can technically be analyzed using any database, meaningful results cannot be always expected and a sanity check must be performed. Both instrumentation and bioinformatic processing in MS-based proteomics are continuously improving at lowering the limit of detection even further. Nevertheless, data output should always be controlled in order to avoid the over-interpretation of results.

## 1. The Mass-Spectrometry-Based Proteomics Workflow and its Limits

The analysis of large sets of proteins, so-called proteomes, took a huge leap forward when mass spectrometry (MS) entered the field shortly after the development of the prized soft ionization techniques, matrix-assisted laser desorption ionization (MALDI) and electrospray ionization (ESI), which enabled the mass measurement of sensitive biomolecules (for their impact on peptide measurements, see ref. [1]). MS has been a driving force in proteomic analysis for more than 20 years and still is, despite the fact that, increasingly, complementary technologies are being introduced to the market. These include antibody- and aptamer-based assays, as well as proximity extension assays (for reviews on technical advances in proteomics, see [2,3]). MS-based experiments rely on high-resolution/high-mass accuracy instrumentation. The technology behind it is constantly being improved and extended, for instance, by increasing the scan speed in order to use the available ions more efficiently or by the inclusion of ion mobility as an additional measurement dimension [3]. Moreover, artificial intelligence and, in particular, deep learning assist at various stages of the proteomics workflow, such as in predicting experimental peptide measurements from amino acid sequences [4].

Once the proteome of a tissue homogenate, biofluid, or cell lysate has been measured using the established workflow of enzymatic protein digestion followed by chromatographic separation (LC) and untargeted high-resolution MS (for proteomics beginners guide, see ref. [5]), the data need to be analyzed by comparing them to databases. These collections contain the sequences for the known proteins of the species from which the sample originated. This means that, e.g., a study of human tissue specimens would use the Uniprot human protein archive, and investigations on laboratory animals such as mice or rats would be based on the entries for these particular species in the Uniprot database. In cases of species that are not heavily studied, their protein sequences may not be available in one of the large public repositories such as Uniprot. In those cases, researchers need to generate such a database themselves or obtain it from colleagues. Only the proteins, which are contained in databases, can subsequently be found by the search algorithm when analyzing the experimental MS data. Thus, the content and the quality of the database are critical.

**Figure 1:**
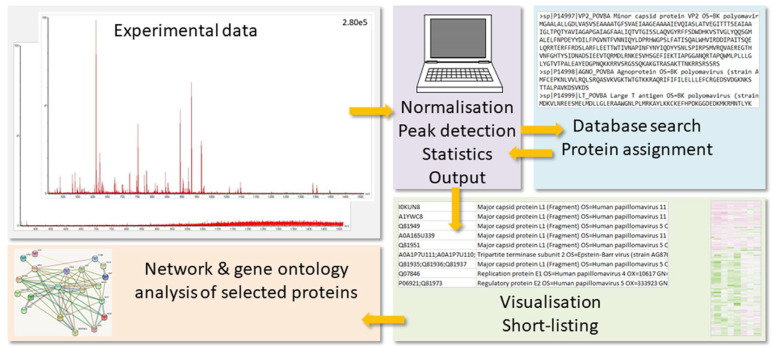
General proteomics workflow. The MS results (low to high quality) need to be prepared for the database search by processes such as data normalization, run alignment, and peak detection (for limits, see text below). The search algorithm uses the mass peaks for the peptide ions and their gas phase fragments to interrogate the protein database for peptide sequences, which would potentially generate the same experimental values. Protein hits are then deduced from the peptide matches. The protein output is subsequently evaluated with respect to significant differences among treatment groups, and short-lists of proteins-of-interest are produced. These are subjected to further evaluation such as pathway, network, and GO analyses.

In Figure 1, the general proteomics workflow is visualized. The MS results need to be prepared for the database search by processes such as run alignment, data normalization, and peak detection. The search algorithm uses the mass peaks for the peptide ions and their gas phase fragments to interrogate the protein database for peptide sequences, which would potentially generate the same experimental values. Protein hits are then deduced from the peptide matches. The protein output is evaluated with respect to significant differences among treatment groups, and short-lists of proteins-of-interest are produced. These are subjected to further evaluation such as pathway, network, and gene ontology (GO) analyses (for a review on bioinformatics analysis, see ref. [6,7]; for a tutorial on best practices for biomarker discovery, see [8]).

Shotgun proteomics experiments are highly amenable to automation because many steps in the workflow can be standardized and rely on computerization. Huge data sets are generated in little time compared to manual MS experiments. In fact, in the in silico processing of proteomics projects, it is mandatory to handle tens of thousands of mass peaks created in a single run. Moreover, low-intensity spectra can be analyzed by software algorithms better than by the human operator, providing the chance of finding low-abundant proteins. On the downside, poor spectra are often the source of false-positive assignments, which are not flagged in a purely computerized experimental approach and passed on to the biologist as solid truth, leading to data over-interpretation.

We currently observe an increasing separation of MS experiments and data analysis and an unfounded excessive trust in automation, which bears dangers regarding the quality of experimental results. This trend has been noticed for some time, and scientists have expressed the need for better control and validation of the results of proteomic analyses (see “Proteomics is analytical chemistry” [9,10], and “A critical review of bottom-up proteomics” [11]). The problem is that MS shotgun experiments produce spectra in a wide range from poor to high quality—from the detection limit of the instrumental setup to its saturation. Mass spectrometers themselves have a dynamic range of approximately four to five orders of magnitude, instrument-, method-, and molecule-depending. For example, for the orbitrap mass analyzer, 5-ppm mass accuracy was reached at a dynamic range of more than 5000, which is about an order of magnitude higher than typical values for time-of-flight instruments [12]. For electrospray instruments’ linear dynamic range, an upper analyte concentration limit of ~10^−5^ molar was reported [13]. In the case of MS imaging, a dynamic range per pixel over 500:1 was obtained from the analysis of tissue sections [14].

**Figure 2:**
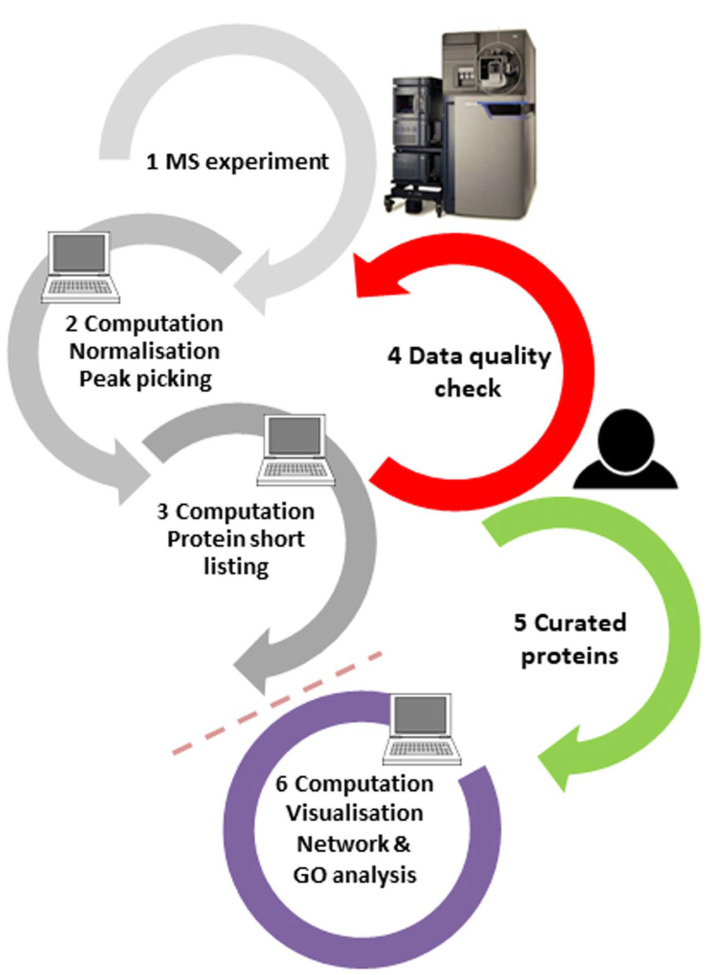
Proteomics experiments are typically performed in the order of steps 1–2–3–6 in an automated manner. Results of purely automatic routines contain, however, false-positive results, which cannot be eliminated simply by changing software processing parameters. Thus, for the—typically few—proteins of most interest, the quality of the peptide spectra responsible for these assignments should be manually checked (step 4) and only reliable hits should be forwarded to further bioinformatics analysis and orthogonal validation (step order 1–2–3–4–5–6). This requires tight interaction of project principal investigators, mass spectrometrists, and bioinformaticians.

Abundant proteins are typically measured with high confidence, but proteins only available at concentrations close to the limit of detection are not, which is evident in the peptide fragmentation spectra. Therefore, we and others have argued before [9,10] that the spectral output should be manually curated, at least for those few proteins which are of topmost interest to the scientist, by adding a quality control step to the workflow (step 4 in Figure 2; for a tutorial on peptide spectral quality, see ref. [10]). For best results, the tight interaction of principal investigators, mass spectrometrists, and bioinformaticians should be ensured in order to iron out any factors impacting the project results, be it from sample preparation, MS settings, or processing filters. Moreover, protein results obtained purely by in silico processes do not have the status of being “identified” according to the criteria of analytical chemistry, and the language in publications should be generally more conservative.

## 2. Database Search

### 2.1. Proteomics of Minor Species

Once experimental MS data are available, they can technically be analyzed by using any database. It is, thus, tempting to screen for proteins of all the species potentially present in the sample. For instance, we have recently investigated human gastric biopsy specimens and serum in an effort to find out biomarkers for gastritis and stomach cancer [15]. Using the Uniprot human database, which is well curated, we detected two marker panels for early and advanced gastric cancer (GC). Subsequently, the question was raised of whether or not microbial proteins could be detected in these samples, because bacteria such as *Helicobacter pylori* are risk factors for GC and can be abundant in patients [16].

**Figure 3:**
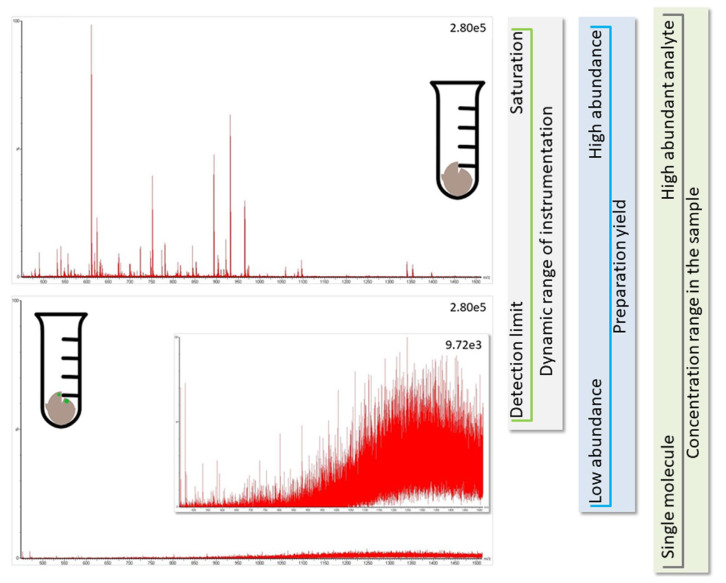
The identification of proteins from other species than the host (green marks in brown bulk material in bottom panel) is challenging because they are likely present at comparatively low concentrations (e.g., the total amount of human protein in infected individual is more than million-fold higher than the available virus material, see Conclusion). The spectra for peptides originating from low abundant proteins (bottom panel showing noise level) are not as informative as those from the major protein species (top panel presenting an informative spectrum). Further, during preparation of the soluble proteome, sample loss occurs, and the LC-MS instrumentation has an experimental window framed by the limit of detection and saturation of LC-column and MS-detector (see vertical side panels for illustration of factors influencing the experimental window). It is, thus, not possible to find all proteins in a sample, and analyte molecules only available with few copies are hardly detected.

However, proteomic experiments provide the best results when the database content matches the proteins expected in the sample; unduly blowing up the database by adding potentially present proteins (e.g., proteomes from other species) only increases the likelihood of chance assignments and, thus, false-positive results. For the same reason, it is not sensible to search for as many protein modifications as possible. In addition, the concentration of the different species, and their respective proteins, in the sample matters. Likely, all species other than the host are present at a comparatively low abundance (Figure 3). From a scientific point of view, it would be best to separate the proteins of the individual species during sample preparation (e.g., by culturing bacteria), but that is often difficult to achieve. Therefore, researchers try to find ways to accommodate this problem during data processing (for approaches, see ref. [16]), but these are all crutches, and their results need to be treated with care.

In our earlier study [16], we have, as a compromise, downloaded the databases for the bacteria from Uniprot and combined them in a single archive rather than searching them individually. This measure was taken to minimize chance assignments of the same mass peaks to different proteins in the various bacteria. We were well aware, and discussed it in the paper, that this analysis had the flaw of not knowing the truly present microbiome, on the one hand, and ignoring the human proteome, on the other hand. Thus, there were bound to be mismatches to microbial proteins, which could have also been explained by human proteins. Therefore, if such analyses are performed, the results should be viewed even more critically than usual. At best, they are a rough test if it is worth it to explore a certain research avenue further by other methods.

### 2.2. Viromics

This is even more true for investigations of the virome in host tissue. It has been known for some time that oncoviruses such as the Epstein–Barr virus (EBV) play a role in GC [17,18,19] and we were, again, facing the question of whether or not our proteomics data could be interrogated for the presence of viral proteins. Viromics is a quickly developing field, but so far, it is based on genomic work [20,21,22,23,24]. Is it sensible, however, to search for viral proteins in samples such as our gastric biopsies [15].

Other than calculation time, such analysis does not require any further resource, so we chose oncogenic viruses based on their involvement in GC [17,18,19] and downloaded their known proteins from Uniprot. The knowledgebase with respect to the protein sequences is diverse, e.g., for the BK virus, only five entries were available; for human papillomavirus HPV16, 9507 entries were available, mostly uncurated. A joint database of all the individual lists could not be used because of system conflicts for unknown reasons. Thus, the data were analyzed versus each individual database. In total, 121 proteins were assigned for EBV and HPVs (for data, see Supplementary Materials and Material and Methods). However, when we tested the topmost hits for their sequence similarity to human proteins, we detected, e.g., 42.7% overlap for regulatory protein E2 of HPV5 (R9QCJ3, Q81976, Q81975) to human protein SRFS2 (Q01130) and 38.5% to SRM2 (I3L182, I3L182). Major capsid protein L1 of HPV5 (R9QCH2, A9JPG1) matched 21.8% to human protein TASOR (Q9UK61). Thus, the sequence homology of viral and human proteins is one major limitation of the experiment.

### 2.3. Low-Intensity Signals

**Figure 4:**
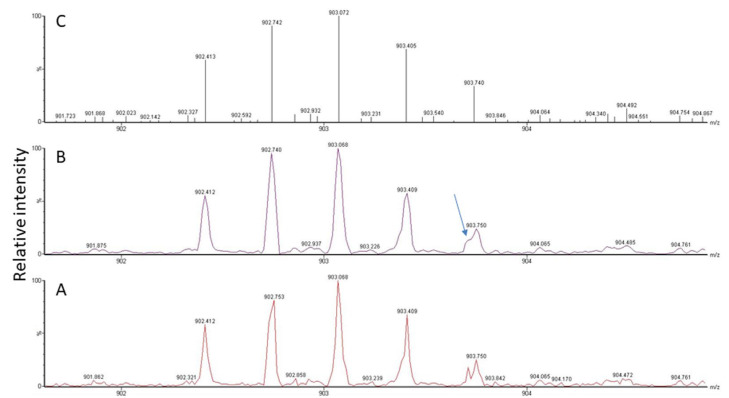
MS signal for a triply-charged peptide detected at *m*/*z* 902.412 in the spectrum (**A**). Raw data (**A**) need to be smoothed (**B**) and centroided (**C**) for subsequent database search. A peptide peak is composed of several isotope peaks, which provide information about the analyte composition based on the natural abundance of individual isotopes in nature. Signals from other peptides may overlap (see arrow), which makes it difficult, depending on instrument resolution, to correctly determine masses, especially at low intensity.

**Figure 5:**
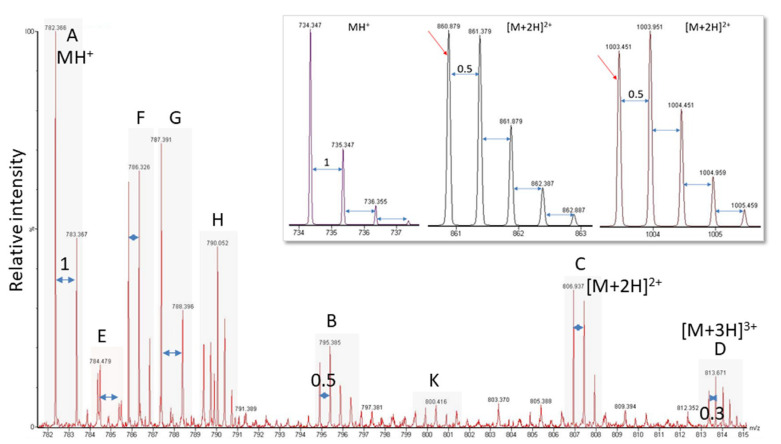
Exemplary electrospray ionization spectrum showing several peptide peaks. (**A**,**E**,**G**) Singly-charged; (**B**,**C**,**F**,**K**) doubly-charged; (**D**) triply-charged; (**H**) overlapping peaks hard to distinguish. Labels and peak distances on the isotope clusters visualize the charge. The inset shows for a singly- (MH^+^) and two doubly-charged ([M + 2H]^2+^) ions that with increasing peptide mass, the monoisotopic mass is not the largest signal of the isotope cluster anymore. The switch occurs at about 1900 Da.

Another problem is the fact that viral proteins do not dominate the sample proteome and their spectral representation is expected to be poor. Though peak detection works perfectly well for spectra of high-to-medium quality, it has difficulties with signals present at close to noise level (for an example, see Figure 3, bottom panel). As is illustrated in Figure 4, the software needs to recognize the isotope pattern of a peak, smooth it in such a way that no information is lost (see treatment of adjacent peak in Figure 4B, marked by arrow), and calculate the centroid mass (Figure 4C) for use in the subsequent database search. The outcome of this process is dependent on instrument resolution with high-resolution mass spectrometers better at resolving adjacent peaks than low-resolution instruments (see Figure 5, peaks in block E). The example spectrum in Figure 5 furthermore shows that it is difficult even for the trained eye to determine the monoisotopic mass for a peak measured close to noise level such as the peak labeled K of a doubly-charged peptide. Software may assign the wrong peak for the peptide mass as a consequence. Confident peak detection can clearly only be achieved for well-defined peaks such as the singly- (A, G), doubly- (B, C, F), and triply-charged (D) ions depicted in Figure 5, although both the human operator and the computer will have difficulties in sorting out peak cluster H. It appears to be an overlap of singly- and doubly-charged, and possibly even triply-charged ions, so the respective monoisotopic masses are hard to establish. In that context, it is important to know that the isotope profile changes with peptide mass; at about 1900 Da, the monoisotopic mass peak is not the largest signal in the isotope profile anymore (see inset in Figure 5).

### 2.4. Ambiguous Spectra

**Figure 6:**
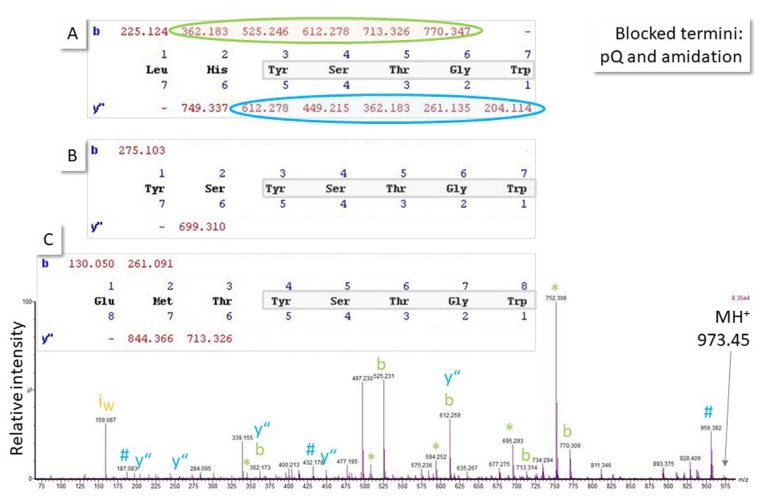
Fragmentation (MS/MS) spectrum of the singly-charged ion of an unknown octapeptide obtained with quadrupole time-of-flight MS. Because it is known from the biological context that both termini could be blocked (C-terminal amidation, N-terminal pyroglutamic acid pQ), at least three sequence hypotheses explain the major peaks in the spectrum (note: pQ in (**A**,**B**); amidation in (**A**–**C**)) sharing the five N-terminal amino acid residues and, thus, the major ions in the spectrum (b- and y”-ion series, ammonia # and water neutral losses *, immonium ion i). As a result of the particular ionization and fragmentation behavior of that peptide, spectral evidence to explain the N-terminal end of the molecule is not convincing and, thus, assignment remains ambiguous until further experimental data from orthogonal methods or measurement of the respective synthetic peptides can support a hypothesis. Accurate mass measurement can also help if a mass spectrometer with sufficient resolution and mass accuracy is available (molecular weight sequence (**A**): 972.445 Da, (**B**): 972.398 Da, (**C**): 972.401 Da). Ion series were calculated using MassLynx software, which treats the N-terminal pQ as a terminus rather than an amino acid residue and, thus, does not mention it in the sequence calculation output shown for (**A**–**C**).

Peptide identification in proteomics is based on peptide fragmentation in the mass spectrometer. The resulting MS/MS spectra are fortunately dominated by backbone fragments and can, thus, be used for sequence elucidation (for tutorial, see ref. [10]). Neutral losses and side chain cleavage also occur. The cleavage outcome is dependent on both the peptide sequence and the fragmentation method, with collision-induced dissociation being mainly used in proteomic workflows. It has been discussed before that spectra of sufficient intensity are necessary for unambiguous sequence assignment [10], but even in cases of high-quality MS/MS spectra, the fragmentation of a peptide may be such that multiple sequence hypotheses can be formulated. The example in Figure 6 shows the spectrum of an unknown, potentially blocked, as known from the biological context, octapeptide where five C-terminal amino acid residues can be assigned with good confidence, but at least three sequence parts can be proposed for the N-terminal residues. This situation is not unusual in MS-based peptide identification, especially because more than 300 posttranslational modifications are known in nature, which cannot all be included by default in the database search because this would blow the search space out of proportion, make room for random peak assignments, and increase the number of false-positive hits.

## 3. Conclusions

Protein identification based on the untargeted analysis of peptide MS spectra is an important procedure in modern day proteomics. Many steps in the workflow have been automatized. However, it is important to understand the spectral information (MS/MS data of peptides) in order to be able to separate reliable data from false-positive assignments. We present a number of caveats for the proteomic analysis of low-abundant species in bulk samples. Without repeating the general limits in proteomics experiments described before [9,10,11], we discuss the feasibility and plausibility of re-processing MS data obtained for, e.g., human biopsies with regard to microbiota and, in particular, viruses.

The analysis of total proteomes has great value in providing an overview of the sample composition, but it is a superficial experiment, which is not always able to properly identify and differentiate homologous proteins or isoforms. Subsequent orthogonal analyses need to clarify the presence of certain proteins of interest. For analyses of minor species in host samples, the limits of the proteomic workflow are amplified because they are present at much lower concentrations in the bulk, resulting in a lower spectral quality for the derived peptides. In fact, it was estimated that each individual infected with SARS-CoV-2 carries about 1–100 billion virions with a total mass of no more than 0.1 mg [25]. Moreover, comprehensive protein sequence databases are not available for every species of potential interest, resulting in a bias in the search results towards known proteins. Database quality, instrumentation, and data processing tools are, however, constantly improving at lowering the limit of detection further [2,3,4]. Nevertheless, it will still be necessary to evaluate proteomics results according to the principles of analytical chemistry.

By way of example, we have processed earlier proteomic data for biopsies from patients with gastroduodenal diseases, including gastric cancer, with respect to viral proteins [12]. Though protein matches for oncogenic viruses were obtained, we urge that they are used only as a case in point rather than as an experimental fact relevant for biology, unless further evidence for the presence of certain proteins has been obtained by other analytical methods.

## 4. Materials and Methods

### 4.1. Patients and Permissions

Extensive details on patients and samples are available in the companion paper to this project (see, ref. [15]). Briefly, symptomatic patients having upper gastroduodenal problems (acid reflux, abdominal pain, heartburn, vomiting, and bloating) attending the Center for Liver & Digestive Diseases, Holy Family Hospital, Rawalpindi, for gastroduodenal endoscopic procedure were enrolled. Biopsies were available from 75 patients and they were divided into groups according to the gastroduodenal clinical manifestations and histopathological evaluation (normal mucosa—NGM (n = 12), mild gastritis—MiG (n = 11), moderate gastritis—MoG (n = 11), marked gastritis—MaG (n = 11), pan gastritis—PanG (n = 5), ulceration—U (gastric ulcer, duodenal ulcer, n = 12), GC (first and advanced stage, n = 13)). Most of the study participants (70%) were *H. pylori*-positive. Gastric biopsy specimens were, if possible, collected from normal (N) and adjacent diseased (D) parts of the stomach antrum during gastroduodenal endoscopy.

Ethical approvals were obtained from the Ethical Technical Committee of the Pakistan Institute of Nuclear Science and Technology (PINSTECH), Islamabad (Ref.-No. PINST/DC-26/2017); the Bioethics Committee of Quaid-i-Azam University, Islamabad (Ref.-No. BBC-FBS-QAU2019-159); the Institutional Research Forum of Rawalpindi Medical University, Rawalpindi (Ref.-No. R-40/RMU); and the Ethics Committee of the University of Münster, Germany (Ref.-No. 2021-339-f-N). Informed written consent was obtained from each participant.

### 4.2. Protein Expression Analysis

Sample preparation and measurements were described in reference [15]. Briefly, proteins were extracted from the gastric biopsies, reduced, alkylated, tryptically digested, and subjected to reversed-phase liquid chromatography (LC) coupled to high-definition MS with Synapt G2 Si/M-Class nanoUPLC (Waters Corp., Manchester, UK). Label-free data-independent quantification experiments were analyzed with Progenesis for Proteomics (QIP, Nonlinear Diagnostics/Waters Corp.) using the Uniprot databases (download 9/6/2022) for varicella zoster virus VZVD (69 entries), JC polyoma virus POVJC (4464 entries), herpes simplex virus 1 HHV11 (446 entries) and 2 HHV2H (74 entries), BK virus POVBA (5 entries), cytomegalovirus HCMVM (325 entries), hepatitis B virus HBVCJ (5 entries), HPV41 (11 entries), HPV1 (143 entries), HPV16 (9507 entries), HPV11 (248 entries), HPV5 (69 entries), HPV4 (24 entries), EBV B95-8 (164 entries), and EBV AG876 (80 entries). At least two peptides were required for protein assignment.

## Data Availability

All data are available in the Supplement.

## Supplementary Materials

The following supporting information can be downloaded at: https://www.mdpi.com/article/10.3390/life13020544/s1, analysis data, Excel table: Supplement.

## Abbreviations

GC—gastric cancer, MiG—mild gastritis, MiGa—atypical MiG, MiGn—normal MiG, MoG—moderate gastritis, MaG—marked gastritis, PanG—pan gastritis, NGM—normal gastric mucosa, HPp—*H. pylori*-positive, HPn—*H. pylori*-negative, D—disease site, N—normal site, MS—mass spectrometry.

## References

[1] Nadler W.M., Waidelich D., Kerner A., Hanke S., Berg R., Trumpp A., Rösli C. (2017). MALDI versus ESI: The impact of the ion source on peptide identification. J. Proteome Res..

[2] Cui M., Cheng C., Zhang L. (2022). High-throughput proteomics: A methodological mini-review. Lab. Investig..

[3] Yates J.R. (2019). Recent technical advances in proteomics. F1000Research.

[4] Mann M., Kumar C., Zeng W.F., Strauss M.T. (2021). Artificial intelligence for proteomics and biomarker discovery. Cell Syst..

[5] Sinha A., Mann M. (2020). A beginner’s guide to mass spectrometry–based proteomics. Biochem.

[6] Schmidt A., Forne I., Imhof A. (2014). Bioinformatic analysis of proteomics data. BMC Systems Biol..

[7] Chen C., Hou J., Tanner J.J., Cheng J. (2020). Bioinformatics methods for mass spectrometry-based proteomics data analysis. Int. J. Mol. Sci..

[8] Nakayasu E.S., Gritsenko M., Piehowski P.D., Gao Y., Orton D.J., Schepmoes A.A., Fillmore T.L., Frohnert B.I., Rewers M., Krischer J.P. (2021). Tutorial: Best practices and considerations for mass-spectrometry-based protein biomarker discovery and validation. Nat. Protoc..

[9] Coorssen J.R., Yergey A.L. (2015). Proteomics is analytical chemistry: Fitness-for-purpose in the application of top-down and bottom-up analyses. Proteomes.

[10] König S. (2021). Spectral quality overrides software score - A brief tutorial on the analysis of peptide fragmentation data for mass spectrometry laymen. J. Mass Spectrom..

[11] Dupree E.J., Jayathirtha M., Yorkey H., Mihasan M., Petre B.A., Darie C.C. (2020). A critical review of bottom-up proteomics: The good, the bad, and the future of this field. Proteomes.

[12] Makarov A., Denisov E., Lange O., Horning S. (2006). Dynamic range of mass accuracy in LTQ orbitrap hybrid mass spectrometer. J. Am. Soc. Mass Spectrom..

[13] Tang K., Page J.S., Smith R.D. (2004). Charge competition and the linear dynamic range of detection in electrospray ionization mass spectrometry. J. Am. Soc. Mass Spectrom..

[14] Bowman A.P., Blakney G.T., Hendrickson C.L., Ellis S.R., Heeren R.M.A., Smith D.F. (2020). Ultra-high mass resolving power, mass accuracy, and dynamic range MALDI mass spectrometry imaging by 21-T FT-ICR MS. Anal. Chem..

[15] Aziz S., Rasheed F., Zahra R., König S. (2022). Gastric cancer pre-stage detection and early diagnosis of gastritis using serum protein signatures. Molecules.

[16] Aziz S., Rasheed F., Zahra R., Akhter T.S., König S. (2022). Microbial proteins in stomach biopsies associated with gastritis, ulcer, and gastric cancer. Molecules.

[17] Wang H., Chen X.L., Liu K., Bai D., Zhang W.H., Chen X.Z., Hu J.K. (2020). SIGES research group. Associations between gastric cancer risk and virus infection other than Epstein-Barr Virus: A systematic review and meta-analysis based on epidemiological studies. Clin. Transl. Gastroenterol..

[18] Takada K. (2000). Epstein-Barr virus and gastric carcinoma. J. Clin. Pathol. Mol. Pathol..

[19] Firoz A., Ali H.M., Rehman S., Rather I.A. (2022). Gastric cancer and viruses: A fine line between friend or foe. Vaccines.

[20] Ramamurthy M., Sankar S., Kannangai R., Nandagopal B., Sridharan G. (2017). Application of viromics: A new approach to the understanding of viral infections in humans. Virusdisease.

[21] Bikel S., Valdez-Lara A., Cornejo-Granados F., Rico K., Canizales-Quinteros S., Soberón X., Del Pozo-Yauner L., Ochoa-Leyva A. (2015). Combining metagenomics, metatranscriptomics and viromics to explore novel microbial interactions: Towards a systems-level understanding of human microbiome. Comput. Struct. Biotechnol. J..

[22] Santiago-Rodriguez T.M., Hollister E.B. (2019). Human virome and disease: High-throughput sequencing for virus discovery, identification of phage-bacteria dysbiosis and development of therapeutic approaches with emphasis on the human gut. Viruses.

[23] Hillary L.S., Adriaenssens E.M., Jones D.L., McDonald J.E. (2022). RNA-viromics reveals diverse communities of soil RNA viruses with the potential to affect grassland ecosystems across multiple trophic levels. ISME Commun..

[24] Special Issue “Viromics: Approaches, advances and applications”. Viruses 2019. https://www.mdpi.com/journal/viruses/special_issues/viromics.

[25] Sender R., Bar-On Y.M., Gleizer S., Bernstein B., Flomholz A., Phillips R., Milo R. (2021). The total number and mass of SARS-CoV-2 virions. Proc. Natl. Acad. Sci. USA.

